# Comparison of GPT-5 and GPT-4o in Solving the Polish Centre for Medical Examinations (CEM) Gastroenterology Examination

**DOI:** 10.7759/cureus.102497

**Published:** 2026-01-28

**Authors:** Wojciech Jaworski, Tomasz Dolata, Piotr Sawina, Ada Latkowska, Melania Olender, Aleksandra Wielochowska, Dawid Boczkowski, Dominika Radej, Anna Kowalczyk, Weronika Majchrowicz, Michalina Loson, Maja Kruplewicz, Aleksandra Stachowicz, Maria Kubiak, Patrycja Dadynska

**Affiliations:** 1 Medicine, Independent Provincial Integrated Public Hospital in Szczecin, Szczecin, POL; 2 Internal Medicine, Multispecialty Independent Public Health Care Institution Hospital in Nowa Sól, Nowa Sól, POL; 3 Internal Medicine, Non-Public Health Care Institution (NZOZ) Hospital in Dzierżoniów, Dzierżoniów, POL; 4 Faculty of Medicine, Wroclaw Medical University, Wrocław, POL; 5 Medicine, Central Teaching Hospital of the Medical University of Lodz, Lodz, POL; 6 Internal Medicine, Central Teaching Hospital of the Medical University of Lodz, Lodz, POL; 7 Plastic Surgery, Department of General and Vascular Surgery, Specialist Medical Center, Polanica-Zdrój, POL; 8 Faculty of Medicine, University of Opole, Opole, POL; 9 Dentistry, Dental Office, Dentistry in the Tenement, Gryfino, POL; 10 Internal Medicine, Independent Provincial Integrated Public Hospital in Szczecin, Szczecin, POL; 11 Internal Medicine, Karol Marcinkowski University Hospital, Zielona Góra, POL

**Keywords:** artificial intelligence (ai), chatgpt-4o, chatgpt-5, final medical examination, gastroenterology medicine, gpt-4o, gpt-5

## Abstract

Introduction: Large language models (LLMs) are increasingly explored as tools for medical education and assessment. While prior studies have demonstrated strong performance of LLMs on undergraduate and general medical examinations, their reliability and calibration on specialty-level certification exams remain insufficiently characterized. In particular, little is known about how model-reported confidence aligns with correctness in high-stakes medical testing.

Objective: The aim of this study was to compare the accuracy of responses and the calibration of self-reported confidence of GPT-4o and GPT-5 when completing a national specialty-level gastroenterology examination administered by the Polish Centre for Medical Examinations (CEM). The CEM gastroenterology exam was selected as a standardized, high-stakes certification assessment that evaluates advanced specialist knowledge and complex clinical problem-solving within a narrowly defined medical domain. Although previous studies have examined the performance of LLMs in other national and international medical examinations, the performance of LLMs in the Polish specialty examination in gastroenterology has not been previously analyzed as a distinct domain. This study, therefore, aims to assess how contemporary LLMs perform within the context of national postgraduate specialty certification and to provide a reference point for comparison with results obtained in other specialties and examination systems.

Methods: Both models were administered 120 multiple-choice questions from the official CEM gastroenterology State Specialization Examination (PES). Accuracy was assessed against the official answer key, with 95% CI calculated using the Wilson method. Paired differences in accuracy were analyzed using McNemar’s test. Self-reported confidence levels were recorded on a 10-point scale, and point-biserial correlations were used to evaluate the relationship between confidence and correctness, with Bonferroni correction applied for multiple testing.

Results: GPT-4o achieved an accuracy of 85.0% (102/120; 95% CI: 77.6-90.3), while GPT-5 achieved 86.7% (104/120; 95% CI: 79.5-91.6). The difference in accuracy was not statistically significant (χ² = 1.0, p = 0.625). Mean confidence levels were similarly high for both models. The confidence-accuracy correlation was weak and non-significant for GPT-4o (r = 0.14), whereas GPT-5 demonstrated a statistically significant positive correlation (r = 0.28), which remained significant after correction for multiple testing.

Conclusions: Both GPT-4o and GPT-5 exceeded the passing threshold for the CEM gastroenterology examination, demonstrating strong performance on a specialty-level medical assessment. Although overall accuracy was comparable, GPT-5 showed superior alignment between confidence and correctness, suggesting improved metacognitive reliability rather than a substantial gain in raw accuracy. These findings highlight the potential educational value of newer LLMs while underscoring important limitations, including the restricted sample size, exam-specific context, and lack of assessment of real-world clinical reasoning. Ethical considerations such as hallucinations, overconfidence, and inappropriate clinical reliance remain critical barriers to direct clinical deployment. Future research should focus on broader exam representativeness, task difficulty stratification, and controlled integration of LLMs into postgraduate medical education.

## Introduction

Artificial intelligence (AI) is increasingly integrated into healthcare, transforming not only clinical practice but also medical education and professional assessment. Among the most influential innovations are large language models (LLMs), which can process complex information, reason through clinical scenarios, and support decision-making. Recent developments have brought two notable models to the forefront: GPT-4o, recognized for its multifunctional capabilities, and GPT-5, the latest generation of OpenAI's (San Francisco, CA) LLMs, featuring enhanced reasoning depth and improved contextual understanding.

Although both models are designed to process and analyze medical knowledge, differences in their architectures and training approaches may influence performance on specialized examinations. Evaluating these models within the framework of a standardized, high-stakes certification exam offers a unique opportunity to examine not only their raw accuracy but also their potential to emulate clinical reasoning in advanced medical contexts [1].

This study focuses on the gastroenterology certification exam administered by the Polish Centre for Medical Examinations (CEM), a national, high-stakes test assessing specialist-level knowledge in disorders of the gastrointestinal tract and complex clinical problem-solving. The exam was chosen because it represents a rigorous benchmark for evaluating advanced clinical reasoning within gastroenterology, going beyond simple recall of factual knowledge, and provides relevant insights for postgraduate medical training and professional assessment [2,3].

Our work extends prior research comparing GPT-4.0 and GPT-3.5 in the Polish Final Medical Examination (LEK), which focused on general medical knowledge at the end of undergraduate training [3]. In contrast, we directly compare GPT-4o and GPT-5 on a specialist-level examination, assessing both performance and the relationship between model confidence and answer correctness. By doing so, we provide novel evidence on the capabilities of next-generation LLMs in advanced medical domains and their potential implications for medical education and professional certification.

The study aims to evaluate the accuracy of responses and the alignment of model confidence with correctness, offering insight into how generative AI may complement traditional training pathways while highlighting the challenges and limitations that must be considered when integrating AI into high-stakes assessments.

## Materials and methods

The study was conducted between August 7 and August 17, 2025. A total of 120 multiple-choice (A-E) questions from the official spring 2025 gastroenterology State Specialization Examination (PES) published by the Polish Centre for Medical Examinations (CEM) [4] were included. Each question had a single correct answer specified in the official CEM answer key. The test comprised both theoretical questions and clinical case-based items. Two questions were formally excluded from candidate scoring by CEM after the examination due to post-exam concerns regarding their clarity. Since such exclusions occur only after the exam has been completed, the models were not informed of this and answered all 120 questions, thus replicating the conditions faced by examinees. These responses were retained in the analysis to maintain methodological consistency. It should be noted, however, that including these questions may introduce minimal systematic bias, which is discussed in the Limitations section.

Two LLMs were evaluated: GPT-4o and GPT-5, accessed via the ChatGPT web interface (OpenAI, publicly available versions from August 2025). GPT-4o was used in standard chat mode, while GPT-5 was used with the “Thinking” feature enabled, allowing enhanced reasoning. Beyond this difference, default user-end settings were applied for both models, without adjustments to temperature or other generation parameters. Each question was manually transcribed into the models in Polish, following the exact wording and format from the CEM website. Transcription was performed without knowledge of the correct answers to reduce bias. Each model completed all 120 questions in a single dedicated chat session, with GPT-4o and GPT-5 run in separate sessions to avoid information transfer between models.

For each question, models selected the most likely correct answer (A-E) and rated their self-assessed confidence on a numerical scale from 0 (no confidence) to 10 (maximum confidence). Recorded variables included (1) the selected answer, (2) correctness relative to the official key, and (3) declared confidence level. All datasets were independently verified three times by different authors to minimize transcription errors and ensure data integrity.

Statistical analyses included calculation of accuracy, mean confidence scores for correct and incorrect answers, and the correlation between confidence and correctness. Accuracy was assessed against the official answer key, with 95% confidence intervals (CIs) calculated using the Wilson method. Paired differences in accuracy were analyzed using McNemar’s test. Self-reported confidence levels were recorded on a 10-point scale, and point-biserial correlations were used to evaluate the relationship between confidence and correctness, with Bonferroni correction applied for multiple testing. Since each model provided a single response per question, there were no missing, tied, or ambiguous data points. The sample size was determined by the total number of questions in the exam (120), and no a priori power analysis was conducted. Limitations related to sample size and potential detection of small differences between models are discussed in the manuscript.

Statistical analysis

Accuracy for GPT-4o and GPT-5 was calculated as the percentage of correct answers, with 95% confidence intervals (CI) estimated using the Wilson method for binomial proportions. Differences in accuracy between the two models were assessed using McNemar’s test. The effect size for paired binary outcomes was expressed as an odds ratio (OR) with 95% CIs, indicating the direction and magnitude of differences. Self-reported confidence scores were summarized using means and standard deviations. The relationship between confidence and correctness was evaluated using point-biserial correlation coefficients, with 95% CIs estimated via bootstrapping (1,000 resamples). Effect sizes were interpreted as small, moderate, or large according to standard conventions. To account for multiple comparisons, p-values from correlation analyses were adjusted using the Bonferroni method. Since both models responded to all questions, there were no missing or ambiguous data points. Questions excluded post hoc by CEM were retained in the analysis because the models were unaware of their exclusion. All analyses were performed in Python 3.11 (pandas, scipy, statsmodels) (Python Software Foundation, Wilmington, DE). A two-sided significance threshold of α = 0.05 was set.

## Results

Accuracy of responses and confidence level

Both GPT-4o and GPT-5 demonstrated high overall accuracy on the spring 2025 gastroenterology PES examination, clearly exceeding the official passing threshold of 60%. GPT-4o correctly answered 102 out of 120 questions, corresponding to an accuracy of 85.0% (95% CI: 77.6-90.3), while GPT-5 achieved 104 correct responses, yielding an accuracy of 86.7% (95% CI: 79.5-91.6) (Tables 1, 2). Paired comparison of accuracy between the two models using McNemar’s test did not reveal a statistically significant difference (χ² = 1.0, p = 0.625) (Table 2). The effect size, expressed as an odds ratio, indicated a small and non-significant advantage for GPT-5, confirming that the overall performance of both models was comparable.

**Table 1:** Comparison of GPT-4o and GPT-5 responses to questions from the spring 2025 gastroenterology specialty examination (PES) with declared confidence levels. The table presents the responses of GPT-4o and GPT-5 compared to the correct answers (A-E), based on the official answer key published by the Medical Examination Center (CEM) in Łódź. For each question, both GPT-4o and GPT-5 declared their self-assessed confidence level, which is reported on a scale ranging from 0 to 10 (0/10 - complete uncertainty, 10/10 - absolute certainty) [4]. PES: State Specialization Examination.

Question number	CEM	GPT-4o	GPT-5	Confidence level of GPT-4o	Confidence level of GPT-5
1	D	D	D	9/10	9/10
2	A	A	A	9/10	9/10
3	B	D	D	9/10	9/10
4	A	A	A	10/10	10/10
5	E	E	E	9/10	9/10
6	C	C	C	9/10	9/10
7	E	E	E	10/10	10/10
8	E	E	E	10/10	10/10
9	C	C	C	9/10	9/10
10	A	A	A	9/10	9/10
11	E	A	A	9/10	9/10
12	B	B	B	10/10	9/10
13	A	C	C	10/10	10/10
14	D	D	D	10/10	9/10
15	D	D	D	9/10	8/10
16	C	C	C	10/10	9/10
17	E	A	A	9/10	9/10
18	D	B	B	8/10	8/10
19	E	E	E	9/10	8/10
20	A	A	A	10/10	10/10
21	E	E	E	9/10	8/10
22	D	D	D	10/10	9/10
23	B	B	B	10/10	9/10
24	D	D	D	10/10	10/10
25	B	B	B	10/10	8/10
26	A	E	E	10/10	7/10
27	A	A	A	10/10	10/10
28	C	C	C	10/10	10/10
29	B	B	B	9/10	7/10
30	E	E	E	10/10	10/10
31	B	B	B	10/10	10/10
32	A	A	A	9/10	9/10
33	E	E	E	10/10	10/10
34	D	D	D	9/10	9/10
35	A	A	A	8/10	8/10
36	B	B	B	10/10	10/10
37	E	E	E	10/10	10/10
38	D	D	D	10/10	10/10
39	D	D	D	10/10	10/10
40	E	E	E	10/10	10/10
41	E	E	E	9/10	9/10
42	C	C	C	9/10	9/10
43	D	D	D	8/10	8/10
44	A	A	A	10/10	10/10
45	C	C	C	9/10	10/10
46	C	D	D	9/10	9/10
47	X	A	A	10/10	10/10
48	E	E	E	9/10	9/10
49	A	A	A	10/10	9/10
50	E	E	A	9/10	8/10
51	B	B	B	9/10	9/10
52	B	B	B	10/10	10/10
53	D	D	D	10/10	10/10
54	X	E	E	8/10	8/10
55	D	D	D	9/10	9/10
56	C	C	C	9/10	10/10
57	A	A	A	9/10	10/10
58	C	C	C	10/10	9/10
59	A	A	A	10/10	10/10
60	A	A	A	10/10	10/10
61	A	A	A	10/10	10/10
62	A	A	A	9/10	9/10
63	C	C	C	9/10	9/10
64	D	E	C	9/10	8/10
65	A	A	A	8/10	9/10
66	C	C	C	9/10	9/10
67	C	A	D	9/10	10/10
68	A	B	B	8/10	8/10
69	E	D	D	10/10	10/10
70	E	E	E	9/10	9/10
71	B	B	B	9/10	9/10
72	D	D	D	10/10	10/10
73	C	C	C	9/10	9/10
74	A	A	A	10/10	10/10
75	B	B	B	9/10	9/10
76	B	B	B	9/10	10/10
77	E	E	E	10/10	10/10
78	D	D	D	9/10	10/10
79	E	E	E	10/10	10/10
80	E	E	E	10/10	10/10
81	A	A	A	9/10	10/10
82	D	D	D	10/10	10/10
83	E	D	B	10/10	9/10
84	B	B	B	10/10	9/10
85	C	C	C	9/10	10/10
86	D	D	D	10/10	10/10
87	A	A	A	8/10	9/10
88	E	E	E	10/10	10/10
89	A	A	A	10/10	10/10
90	C	C	C	9/10	10/10
91	E	E	E	10/10	9/10
92	A	A	A	9/10	9/10
93	A	A	A	10/10	10/10
94	E	D	E	9/10	10/10
95	A	A	A	9/10	9/10
96	D	D	D	8/10	9/10
97	D	C	C	10/10	10/10
98	C	D	C	10/10	10/10
99	E	E	E	10/10	10/10
100	B	B	B	10/10	10/10
101	A	A	A	9/10	10/10
102	B	B	B	9/10	10/10
103	E	E	E	9/10	10/10
104	D	D	D	9/10	9/10
105	C	C	C	10/10	10/10
106	A	A	A	9/10	10/10
107	E	E	E	10/10	9/10
108	D	A	D	9/10	9/10
109	B	B	B	10/10	10/10
110	C	C	C	9/10	9/10
111	D	D	D	9/10	9/10
112	B	B	B	10/10	10/10
113	E	E	E	9/10	10/10
114	A	A	A	9/10	10/10
115	C	C	C	10/10	10/10
116	E	E	E	10/10	10/10
117	D	D	D	10/10	10/10
118	E	E	E	9/10	10/10
119	E	E	E	10/10	10/10
120	E	E	E	10/10	10/10

**Table 2:** Accuracy of GPT-4o and GPT-5 on the spring 2025 gastroenterology PES examination (n = 120). Data are presented as absolute numbers and percentages with 95% confidence intervals (CIs) calculated using the Wilson method. Paired comparison between models was performed using McNemar’s test, which did not demonstrate a statistically significant difference in accuracy. PES: State Specialization Examination.

Model	Correct answers (n)	Accuracy (%)	95% CI (Wilson)	χ²	p-value
GPT-4o	102	85.0	77.6–90.3	1.0	0.625
GPT-5	104	86.7	79.5–91.6		

**Figure 1:**
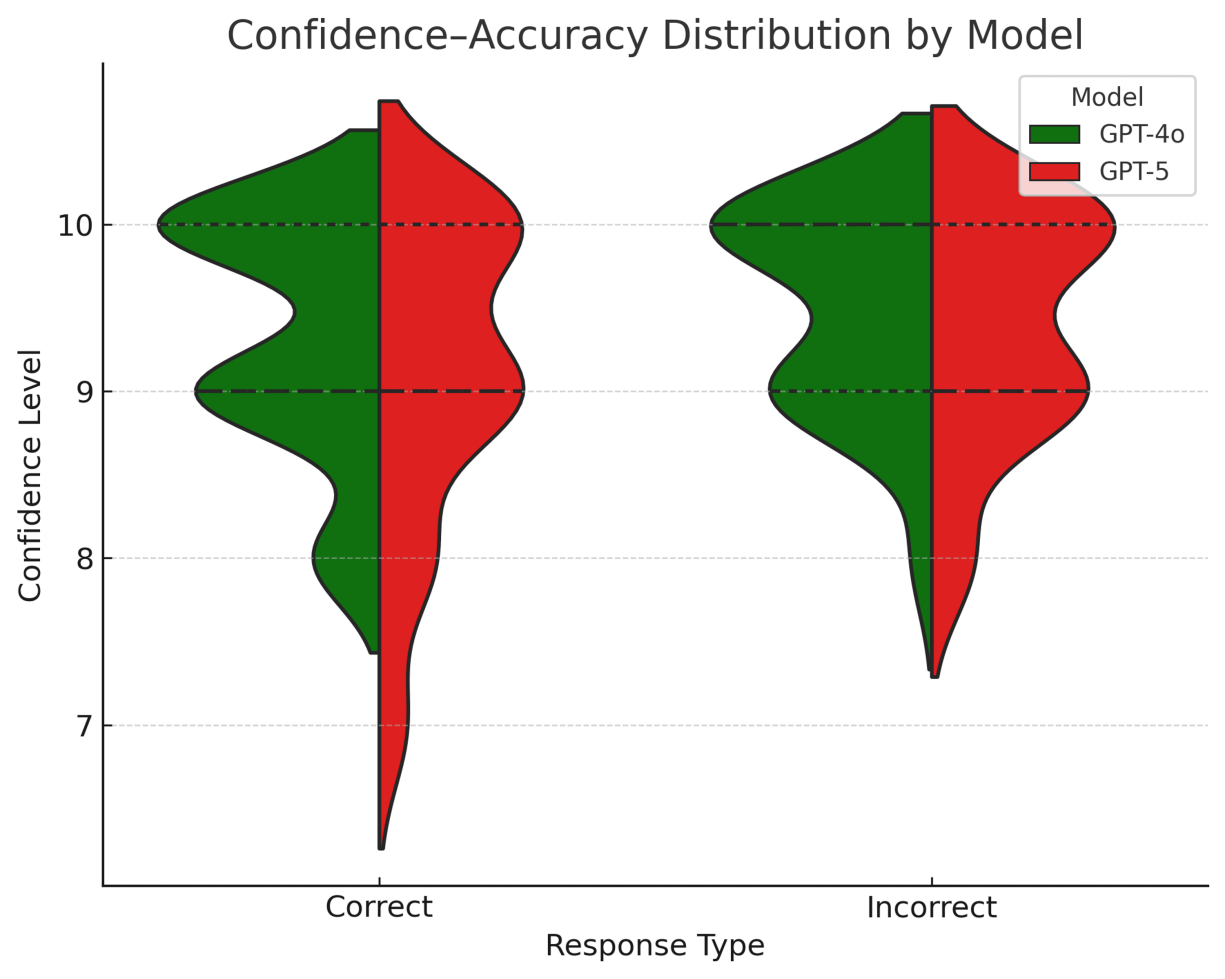
The declared confidence level of both models depending on the accuracy of the answers. The values are presented separately for correct and incorrect responses. Each plot presents the results obtained for GPT-4o and GPT-5.

Self-reported confidence ratings were consistently high for both models, with a pronounced ceiling effect. For GPT-4o, 112 out of 120 responses (93.4%) were rated at 9/10 or 10/10, whereas GPT-5 assigned confidence levels of 9/10 or 10/10 to 107 responses (89.2%) (Table 1 and Figure 1).

The mean confidence score for GPT-4o was 9.425 (SD = 0.6172), compared with 9.40 (SD = 0.7265) for GPT-5 (Table 3), indicating similarly high confidence calibration at the aggregate level (Table 3).

**Table 3:** Comparison of mean confidence and standard deviations between GPT-4o and GPT-5. Data are presented as absolute numbers (n).

Model	Mean confidence	SD (sample)
GPT-4o	9.425	0.6172
GPT-5	9.4	0.7265

Confidence-accuracy correlation

The association between confidence ratings and answer correctness differed between the two models. For GPT-4o, the point-biserial correlation between confidence and correctness was weak and did not reach statistical significance (r = 0.14, 95% CI: −0.05 to 0.31, p = 0.13).

**Figure 2:**
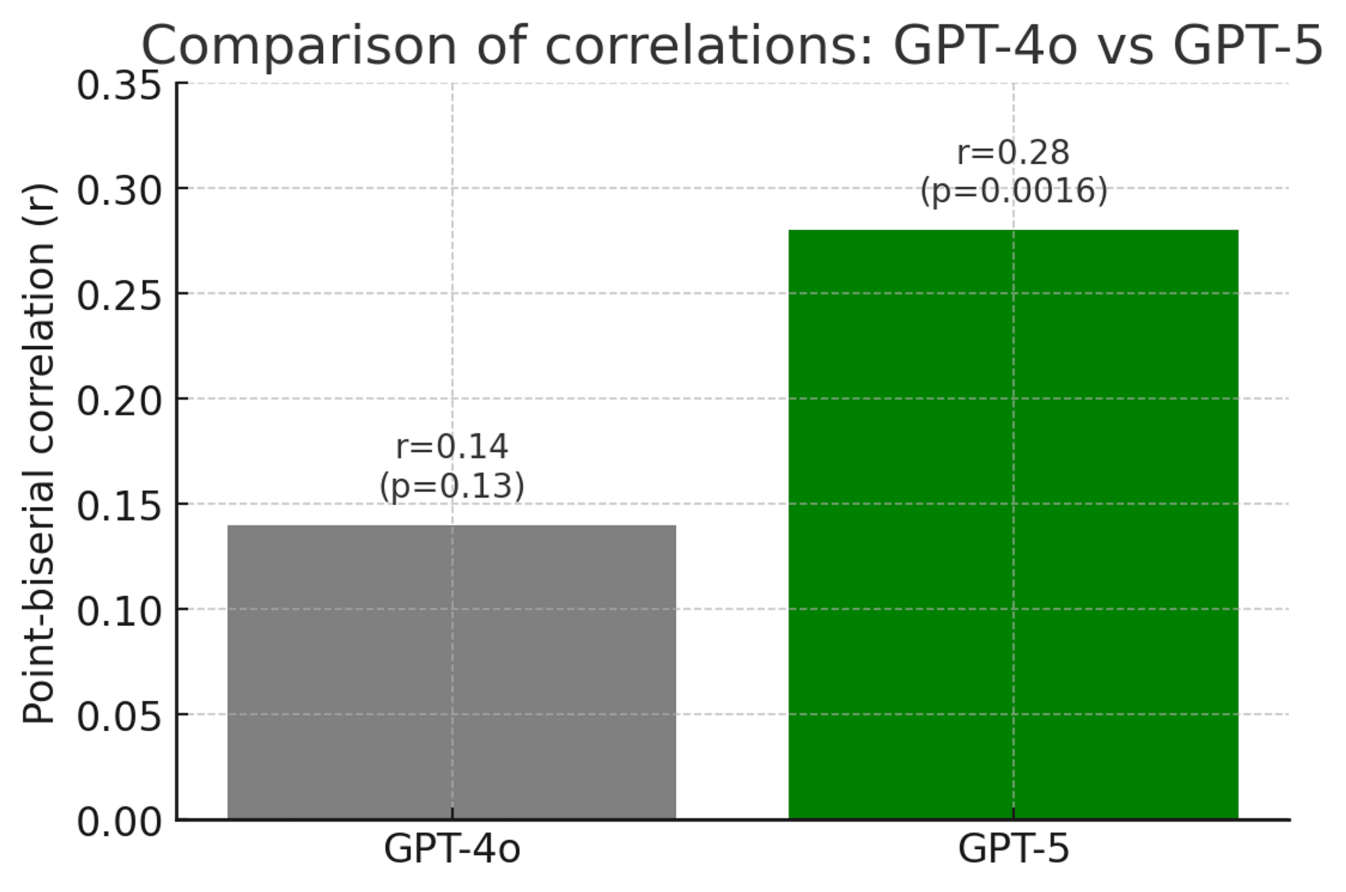
Comparison of GPT-4o and GPT-5 in the correlation between self-reported confidence and correctness of responses on the spring 2025 gastroenterology PES examination. The values are presented separately for GPT-4o and GPT-5. A higher point-biserial correlation (r) score was observed for GPT-5 answers. PES: State Specialization Examination.

In contrast, GPT-5 demonstrated a statistically significant positive correlation between confidence and correctness (r = 0.28, 95% CI: 0.12-0.42, p = 0.0016). After Bonferroni correction for two correlation tests (adjusted α = 0.025), this association remained statistically significant, indicating improved alignment between confidence and performance for GPT-5 (Figure 2).

Model discrepancies

Direct comparison of paired responses showed that GPT-5 provided correct answers in three cases where GPT-4o failed, while GPT-4o outperformed GPT-5 in one case. In 101 instances, both models answered correctly, whereas in 15 instances, both models produced incorrect responses (Table 4).

**Table 4:** Contingency table comparing paired responses of GPT-4o and GPT-5 on the spring 2025 gastroenterology PES examination (n = 120). Data are presented as absolute numbers (n). Subsequent comparative analysis using McNemar’s test did not demonstrate a statistically significant difference in accuracy between the two models (p = 0.625, χ² = 1.0) [4]. PES: State Specialization Examination.

	GPT-5 Incorrect	GPT-5 Correct	p-value	χ² value
GPT-4o Incorrect	15	3	0.625	1
GPT-4o Correct	1	101		

Among the incorrect responses, 12 questions elicited identical incorrect answers from both models, while in three cases, the models selected different incorrect options. The odds ratio derived from the paired comparison indicated a small and non-significant effect size, further supporting the absence of a meaningful difference in overall accuracy between GPT-4o and GPT-5.

## Discussion

This study compared the performance of GPT-4o and GPT-5 on the spring 2025 gastroenterology PES examination administered by the Polish Centre for Medical Examinations. Both models achieved high overall accuracy, well above the official passing threshold of 60%, with GPT-5 obtaining a marginally higher proportion of correct responses. However, the difference in accuracy did not reach statistical significance, indicating broadly comparable performance in terms of correctness.

A notable distinction between the models was observed in their metacognitive calibration. GPT-5 demonstrated a statistically significant positive correlation between confidence and correctness, whereas GPT-4o showed a weaker and non-significant association. This finding suggests that GPT-5 may be more effective in aligning self-assessed certainty with actual performance, a property that is particularly relevant for the safe application of LLMs in clinical decision support. However, both models exhibited pronounced overconfidence across correct and incorrect responses, which remains a major limitation for their use as reliable tools in medical education. Previous work by Omar et al. similarly highlighted systematic overconfidence in contemporary LLMs and emphasized the need for improved calibration before broader clinical adoption [5].

When discrepancies between the two models were examined directly, GPT-5 provided correct answers in only three cases where GPT-4o failed, while GPT-4o outperformed GPT-5 in a single case. This small number of discordant responses limits the interpretability of differences between the models and reinforces the conclusion that their overall performance profiles are highly similar. Moreover, both models failed on the same subset of questions, suggesting that certain examination items pose persistent challenges regardless of model generation.

These findings contrast with earlier studies reporting more substantial performance differences between successive generations of language models. In particular, prior evaluations of medical licensing examinations demonstrated that GPT-4 markedly outperformed GPT-3.5, surpassing official passing thresholds and achieving results closer to those of human candidates [3]. In comparison, the performance gap between GPT-4o and GPT-5 observed in the present study was narrow, suggesting that recent model development may be yielding diminishing returns in terms of accuracy.

An additional observation was that GPT-4o and GPT-5 produced identical incorrect answers for a considerable proportion of failed questions. This convergence of errors implies that certain items require nuanced clinical reasoning or rely on domain-specific knowledge that remains difficult for current LLMs to process. Similar patterns of shared failure modes have been described in diagnostic reasoning benchmarks, where even state-of-the-art models struggled with the same complex cases [6]. At the same time, the role of examination design should not be overlooked, as ambiguous wording or violations of standard item-writing principles may contribute to systematic errors [7].

From an ethical perspective, the integration of generative AI into medical education and clinical decision-making raises important concerns related to autonomy, responsibility, and transparency. As LLMs increasingly influence professional judgment, maintaining human oversight and clear accountability frameworks becomes essential to prevent inappropriate delegation of responsibility [8,9]. Without such safeguards, even highly accurate systems may introduce ethical and legal risks that outweigh their potential benefits.

Limitations

Several limitations of the present study should be acknowledged. All interactions with the models were conducted in Polish, which may have influenced their performance compared to English-based datasets. Two questions that were excluded from scoring by CEM were still analyzed, potentially introducing a minimal systematic bias in accuracy estimates. The study used a fixed sample size of 120 questions, corresponding to the total number of items in the official exam; no a priori power calculations were conducted, which may limit the ability to detect small differences between the two models. Manual transcription of questions introduces a potential source of human error, which was mitigated through a three-step verification process but cannot be entirely excluded. The study was conducted in August 2025, and given the rapid pace of AI development, the results may not generalize to future versions of these models. Although confidence levels were systematically collected, their interpretation remains constrained by the models’ internal algorithms, which may not correspond directly to human-like self-assessment. Reproducibility strategies were limited to documentation of procedures and stepwise verification of all data, but future studies may require additional validation across different sessions and versions.

Additionally, ethical considerations such as data privacy, transparency of model reasoning, and the potential risks of over-reliance on AI in high-stakes examinations should be noted. These factors are important to ensure responsible use of LLMs and to maintain fairness, accountability, and trust in medical certification contexts.

Despite these limitations, this study provides valuable insights into the capabilities of LLMs in medical certification contexts. The observed improvement in confidence calibration of GPT-5 compared to GPT-4o suggests progress toward models that can more reliably gauge their own reliability, which is important if such systems are to be trusted in clinical decision-making and professional education.

## Conclusions

Both GPT-4o and GPT-5 achieved results well above the official passing threshold on the spring 2025 gastroenterology PES, demonstrating that advanced LLMs are capable of handling complex specialty-level assessments. While GPT-5 showed a slight advantage in accuracy and superior calibration of confidence, the overall performance between the two models was highly comparable. The narrow gap between GPT-4o and GPT-5 contrasts with the more substantial differences reported between GPT-3.5 and GPT-4o in earlier studies. These findings should be interpreted in the context of several limitations, including the modest sample size (120 questions), the focus on a single national specialty exam, and potential ambiguities or variable difficulty of certain test items, which may limit generalizability to other exams or real-world clinical tasks. Nevertheless, the results highlight the potential role of generative AI as a complementary tool in postgraduate medical education, for example, as a practice or revision aid, while also underscoring the need for careful evaluation of its limitations and the design of robust oversight strategies before integration into formal assessment and clinical practice.

Consistent with our initial expectations, we hypothesized that GPT-5 would perform at least as well as GPT-4o in terms of accuracy and would exhibit a stronger alignment between confidence and correctness. The results align with this hypothesis, as GPT-5 demonstrated modestly higher accuracy and improved calibration of confidence, suggesting enhanced reliability in assessing its own performance. These findings suggest that advanced LLMs, such as GPT-5, could serve as valuable support tools in specialist-level medical education, provided human oversight is maintained and the limitations of the models are carefully considered. Future research should focus on further developing the metacognitive capabilities of AI and evaluating their performance in more diverse and practical clinical contexts.
